# Development and Validation of a Nomogram to Predict Type 2 Diabetes Mellitus in Overweight and Obese Adults: A Prospective Cohort Study from 82938 Adults in China

**DOI:** 10.1155/2020/8899556

**Published:** 2020-12-07

**Authors:** Qingqing Liu, Jie Yuan, Maerjiaen Bakeyi, Jie Li, Zilong Zhang, Xiaohong Yang, Fangming Gao

**Affiliations:** ^1^Department of Cardiology of People's Hospital of Xinjiang Uygur Autonomous Region, Urumqi, Xinjiang, China; ^2^The First Affiliated Hospital of Xinjiang Medical University, Urumqi, Xinjiang Uygur Autonomous Region, China; ^3^Department of Respiratory and Intensive Care Medicine of People's Hospital of Xinjiang Uygur Autonomous Region, Urumqi, Xinjiang, China

## Abstract

**Background:**

The twin epidemic of overweight/obesity and type 2 diabetes mellitus (T2DM) is a major public health problem globally, especially in China. Overweight/obese adults commonly coexist with T2DM, which is closely related to adverse health outcomes. Therefore, this study aimed to develop risk nomogram of T2DM in Chinese adults with overweight/obesity.

**Methods:**

We used prospective cohort study data for 82938 individuals aged ≥20 years free of T2DM collected between 2010 and 2016 and divided them into a training (*n* = 58056) and a validation set (*n* = 24882). Using the least absolute shrinkage and selection operator (LASSO) regression model in training set, we identified optimized risk factors of T2DM, followed by the establishment of T2DM prediction nomogram. The discriminative ability, calibration, and clinical usefulness of nomogram were assessed. The results were assessed by internal validation in validation set.

**Results:**

Six independent risk factors of T2DM were identified and entered into the nomogram including age, body mass index, fasting plasma glucose, total cholesterol, triglycerides, and family history. The nomogram incorporating these six risk factors showed good discrimination regarding the training set, with a Harrell's concordance index (C-index) of 0.859 [95% confidence interval (CI): 0.850–0.868] and an area under the receiver operating characteristic curve of 0.862 (95% CI: 0.853–0.871). The calibration curves indicated well agreement between the probability as predicted by the nomogram and the actual probability. Decision curve analysis demonstrated that the prediction nomogram was clinically useful. The consistent of findings was confirmed using the validation set.

**Conclusions:**

The nomogram showed accurate prediction for T2DM among Chinese population with overweight and obese and might aid in assessment risk of T2DM.

## 1. Introduction

Globally, type 2 diabetes mellitus (T2DM) is a common public health problem that has affected 422 million adults and caused 1.6 million deaths in 2016 [1, 2]. Furthermore, T2DM causes huge financial burden. The health expenditure of diabetes alone is 673 billion dollars in 2015, accounting for 12% of total expenditure [3]. However, the global burden of disease study and epidemiological studies have confirmed that the prevalence of T2DM has increased rapidly worldwide in the last three decades, especially in developing countries including China [4–6]. China is the world's most populous nation and the largest developing country. Almost one in four of patients with diabetes all over the world lives in China, which makes China become the country with the largest T2DM population in the world [5].

Simultaneously, the prevalence of overweight and obesity all over the world has been increasing steadily over the past several decades [7]. In 2016, World Health Organization (WHO) estimated 39% and 13% of adults (≥18 years) in the world being overweight and obese, respectively [2]. Accumulating surveys indicate overweight/obesity to be a major risk factor for T2DM [8–10]. Previous cohort studies indicate that overweight and obese adults are 2.5 times more likely to develop T2DM than normal weight individuals [11]. Additionally, compared with overweight and obese adults or T2DM alone, patients with T2DM and overweight and obesity have an increased risk of cardiovascular-related mortality [12].

The twin epidemic and parallel escalation of overweight/obesity and T2DM is a major health crisis globally. Approximately 63% of patients with T2DM are overweight or obese in China [13]. Therefore, it is of great significance to distinguish individuals with high risk of suffering from T2DM from those with low risk and follow-up with those high-risk subjects closely for early detection and prevention of T2DM. Though several prediction models were established for diabetes [14, 15], traditional risk factors related to T2DM might play a different role in overweight and obese adults. In addition, most of the predictive score models are built in European and American populations [16, 17], which may not be suitable for the prediction in Chinese population. Moreover, some prediction models built in the general population might underestimate T2DM risk in overweight and obese adults. To our knowledge, a prediction model has not been developed specifically to predict T2DM in overweight and obese population.

Accordingly, our study aimed to establish and validate a comprehensive visual predictive model for T2DM in Chinese with overweight and obesity. The proposed nomogram can help healthcare workers and individuals assess the risk of T2DM, thus promoting early detection and intervention for T2DM.

## 2. Methods and Materials

### 2.1. Setting and Participants

Data for this study were obtained from a prospective cohort study which was established by the Rich Healthcare Group in China from 2010 to 2016. It is a computerized database including all medical records for participants who received a health check. This cohort study was conducted in 43 sites across 11 provinces involving 685277 participants. The number of subjects with duration of follow-up more than two years was 225575 participants. Finally, a total of 211833 participants free of diabetes at baseline were included in the cohort study. This study is a secondary data analysis of the cohort data which was downloaded from a shared database by Chen et al. [18, 19] in the Dryad Digital Repository (http://www.datadryad.org). We analyzed the data for 82938 overweight and obese participants in this current paper.

### 2.2. Data Collection

Trained staff used standardized electronic questionnaires and collected data on demographic characteristics (age, gender) and health-related behaviors (alcohol consumption and cigarette smoking) in each visit to the health check center. Blood pressure (BP) of each participant was measured using the uniform sphygmomanometer. Height and weight were also measured to the nearest 0.1 cm and 0.1 kg, respectively, by trained staff. Body mass index (BMI) was derived as weight divided by the square of height (kg/m^2^).

Fasting for ≥10 h venous blood samples was collected for all participants. Then, fasting plasma glucose (FPG), total cholesterol (TC), triglycerides (TG), low-density lipoprotein cholesterol (LDL-C), high-density lipoprotein cholesterol (HDL-C), alanine aminotransferase (ALT), aspartate aminotransferase (AST), blood urea nitrogen (BUN), and concentration of creatinine (CCR) were tested at local health check center using an autoanalyzer.

The variables for each case were extracted from the raw data as follows: age, gender, sites, height, weight, BMI, FPG, systolic and diastolic BP (SBP, DBP), TC, TG, LDL-C, HDL-C, ALT, AST, BUN, CCR, smoking and alcohol consumption status, family history of diabetes, years of follow-up, and eventual diagnosis of diabetes.

### 2.3. Follow-Up Data Collection

The annual health check of the participants was considered as a follow-up examination. The primary outcome measure was the first (incident) diagnosis of T2DM, which was recorded on the general practice computer records. FPG and lipids profiles and presence of T2DM were evaluated as per baseline. Diabetes was defined as FPG ≥7.0 mmol/L or a self-reported presence of T2DM. If a participant developed diabetes during follow-up, the participant was asked in detail when the diabetes occurred, to the exact date of month.

### 2.4. Definition

Overweight and obesity were classified if BMI is between 24.0 and 27.9 Kg/m^2^ and ≥28.0 Kg/m^2^, respectively [20]. Current cigarette smoking was coded as yes/no. Current alcohol drinking was defined as yes/no. Family history of diabetes was categorized into yes/no. Hypertension is defined as SBP ≥140 mmHg and/or DBP ≥90 mmHg. Dyslipidemia was defined as a combination of one or more statuses: TC ≥ 6.22 mmol/L, LDL-C ≥ 4.14 mmol/L, HDL-C < 1.04 mmol/L, and TG ≥ 2.26 mmol/L in terms of criteria recommended by Chinese guidelines for the Prevention and treatment of dyslipidemia in adults [21].

### 2.5. Statistical Analysis

Descriptive analyses were conducted for 82938 participants using SPSS 20.0 for Windows (SPSS Inc., Chicago, IL). All continuous variables were summarized as means ± standard deviations (M ± SD), and categorical variables were expressed as frequency (*n*) and proportions (%), and the results were compared using Student's *t*-test and the chi-square test to detect the statistical significances, respectively.

The developement and the assessment of nomogram were divided into four steps. First, we randomly selected 70% of the participants (*n* = 58056) as training set to construct the model. We reserved the remaining 30% (*n* = 24882) as validation set for validation. Second, we identified independent predictive features using nonzero coefficients in the least absolute shrinkage and selection operator (LASSO) regression model [22, 23]. Third, Cox proportional hazards model was applied to construct a predicting nomogram based on the selected feature from the LASSO regression model [24], with results presented as hazards ratio (HR) with associated 95% confidence interval (95% CI) and corresponding *p* value. Fourth, the discrimination and calibration of the nomogram were assessed by Harrell's concordance index (C-index) and the area under the receiver operating characteristic curve (AUC) and calibration curves plot, respectively [25, 26]. Finally, to quantify the net benefits at different threshold probabilities in the model, decision curve analysis (DCA) was conducted to determine the usefulness of the nomogram in the validation cohort [27]. The nomogram and the bootstrap analysis were performed using the package of “rms” in *R* version 3.5.1. A *p* value < 0.05 was considered to indicate significance.

## 3. Results

### 3.1. Baseline Characteristics

In total, 82938 overweight and obese subjects with mean age 44.99 ± 12.98 years were enrolled with men accounting for 72.3%. The median follow-up time for all participants in this study was 2.98 years (range: 2.15–3.93 years). During the follow-up period in this study, the overall incidence of T2DM was 3.7% (*n* = 3069). There were no significant differences between the training set and the validation set for baseline characteristics excepted gender and smoking status (*p* range:0.056 to 0.943) (Table 1).

### 3.2. Predicted Feature Selection

**Figure 1:**
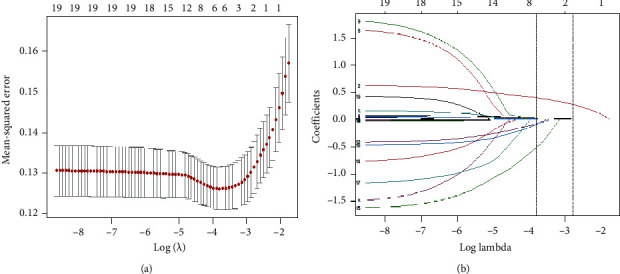
Variable selection using the LASSO binary regression model. Notes: optimal parameter (lambda) selection in the LASSO model used tenfold cross-validation via minimum criteria. The partial likelihood deviance (binomial deviance) curve was plotted versus log (lambda). Dotted vertical lines were drawn at the optimal values by using the minimum criteria and the 1-SE of the minimum criteria (the 1-SE criteria). LASSO coefficient profiles of the 19 features. A coefficient profile plot was produced against the log (lambda) sequence. Vertical line was drawn at the value selected using tenfold cross-validation, where optimal lambda resulted in six features with nonzero coefficients. LASSO, least absolute shrinkage and selection operator; SE, standard error.

We used the LASSO regression model to screen independent predicting features of T2DM in training set. Six potential predictors were screened out of 19 factors in the study (∼3 : 1 ratio; Figures 1(a) and 1(b)) and were with nonzero coefficients (min lambda of 0.02238) in the LASSO regression model. These factors included age, BMI, FPG, TC, TG, and family history which were presented in Table 2.

### 3.3. Construction and Assessment of Nomogram

**Figure 2:**
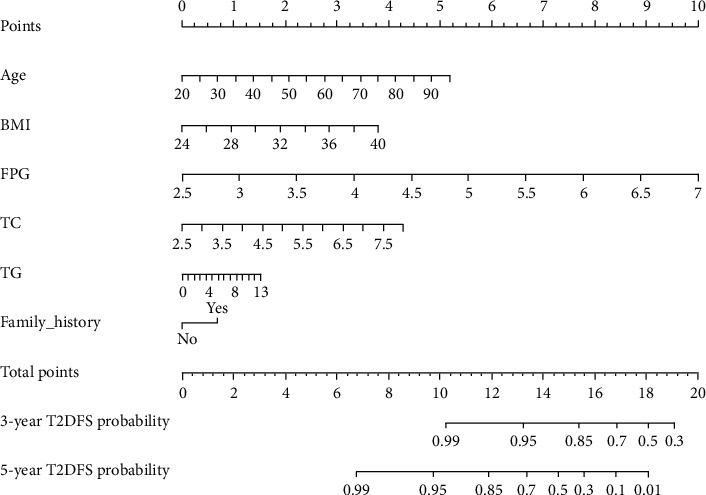
Nomogram to predict 3- and 5-year T2DFS probability for overweight/obese population. T2DFS, type2 diabetes-free survival; BMI, body mass index; FPG, fasting plasma glucose; TC, total cholesterol; TG, triglycerides.

**Figure 3:**
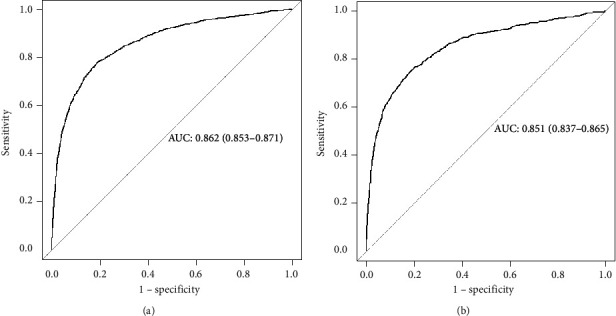
The receiver operating characteristic curve of nomogram. (a) In training set; (b) in validation set.

**Figure 4:**
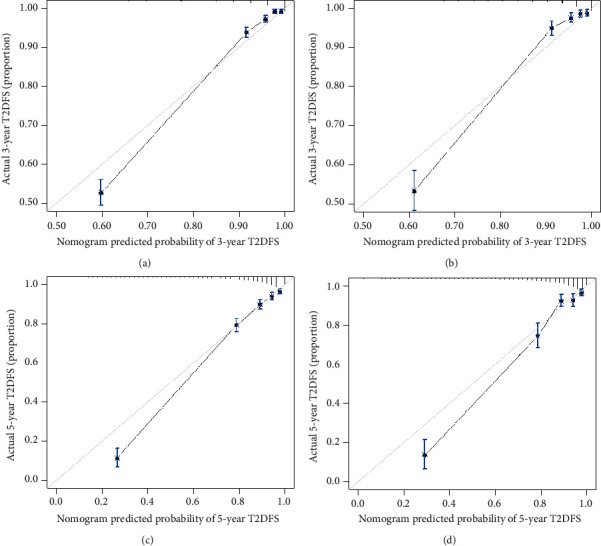
Calibration curves of the nomogram prediction in the study. The observed T2DFS is shown compared with the nomogram at 3 years (a) and 5 years (c) using the training set and validation set (b and d), respectively. Notes: the *x*-axis represents the predicted T2DFS probability. The *y*-axis represents the actual identified subjects with T2DFS. The diagonal gray line represents a perfect prediction by an ideal model. The solid line represents the performance of the nomogram, of which a closer fit to the diagonal gray line represents a better prediction.

The predictive nomogram that integrated all the significant features for the type 2 diabetes-free survival (T2DFS) probability was then developed (Figure 2). The C-index and AUC for the predictive nomogram was 0.859 (95% CI: 0.850–0.868) and 0.862 (95% CI: 0.853–0.871), respectively, which indicated the model's good discrimination (Figure 3(a)). The calibration of nomogram for the T2DFS probability at 3 and 5 years demonstrated good agreement by performing the calibration curve plot (Figures 4(a) and 4(c)).

### 3.4. Internal Validation of the Nomogram

The nomogram showed good discrimination with a C-index of 0.848 (95% CI: 0.833–0.863) and AUC of 0.851 (95% CI: 0.837–0.865) through internal validation in the validation set. Additionally, the good calibration of the prediction nomogram was confirmed in the validation set (Figures 4(b) and 4(d)). Thus, this prediction nomogram performed well using both the training and validation sets.

### 3.5. Clinical Use of Nomogram

**Figure 5:**
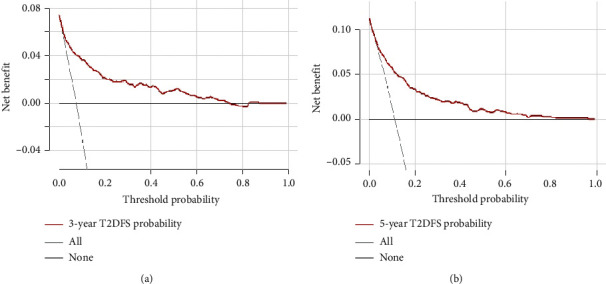
Decision curve analysis for the nomogram for predicting T2DFS in validation set. Notes: decision curves for T2DFS at 3 years (a) and 5 years (b) were applied to the nomogram. The *y*-axis measures the net benefit. The red line represents the nomogram. The thin solid line represents the assumption that all subjects are non-T2DFS. Thin thick solid line represents the assumption that all subjects are T2DFS.

The DCA for the nomogram showed that when the threshold T2DFS in overweight and obese adults ranged between 3.9% and 73.5% at 3 years and between 5.1% and 82.3% at 5 years, using this nomogram to predict the T2DFS probability yielded more net benefit than the scheme, which showed the nomogram to be clinically useful (Figure 5).

## 4. Discussion

Evidence is mounting that high BMI causes the incidence of T2DM [8–10]. Coexistence of obesity/overweight and T2DM is associated with increased risk of stroke, angina, and coronary heart disease and constitutes a significant cardiovascular health burden [12]. Primary prevention and timely intervention are at the core of preventing or postponing onset of T2DM. Therefore, early identification of those individuals at high risk of developing diabetes in overweight and obese adults is vital for reducing the incidence. Accordingly, we attempted to develop and validate a nomogram to predict the T2DFS probability at 3 and 5 years in Chinese with overweight and obesity.

The nomogram developed is simple (consisting of only six factors, during selection of variables for each block, many were eliminated because they were not associated with T2DM or because they showed strong colinearity with other variables) and shows good standardization and ability to discriminate. It is worth mentioning its high sensitivity (approximately 90%), indicating that the factors included are capable, as a whole, of predicting properly the risk of developing T2DM in overweight and obese adults.

T2DM is the ninth cause of disease burden worldwide [4]. Therefore, several researchers have constructed T2DM risk prediction scores [14–17]. However, there are racial and ethnic differences in the prediction factors of T2DM since environmental and genetic characteristics differ among various racial/ethnic populations [28]. Consequently, T2DM risk assessment model developed in white populations are not suitable for Chinese population [14, 15, 29]. Moreover, several predicting models might not accurately predict the future risk of T2DM because they are based on participant coming from single study site, cross-sectional studies, or on relatively small sample size [30–32]. In addition, though there are several models based on Asian or Chinese, they did not contain some of other significant risk factors such as blood lipid levels and family history of diabetes, which might result in insufficient accuracy with small AUC of model [32, 33]. Furthermore, the T2DM risk prediction scores developed in the general population cannot accurately predict the risk of T2DM in overweight and obese adults. To our knowledge, current study is the first to develop and validate a predicted nomogram for predicting 3-year and 5-year incidence probability of T2DFS in a Chinese population with overweight and obesity based on multicenter cohort study. Our model shows good accuracy and excellent agreement in training and validation set, which suggests that it contains good transportability and generalizability.

Results of the current study show that the risk factors related to T2DM in overweight and obese adults include age, BMI, FPG, TC, TG, and family history of diabetes. This is consistent with the previous studies reporting the risk factors of T2DM [8, 34–36]. Currently, the mechanism on older people prone to develop T2DM might be attributed to aging *β*-cells with lower glucose responsiveness and glucose sensitivity and age-related islet cell DNA methylation, which affects insulin secretion and causes T2DM [35, 37]. High BMI has been widely known as one of major risk factors for T2DM. It commonly coexist with T2DM. Our study shows that BMI also plays an important role in incidence of T2DM, which is related to insulin resistance derived from high BMI inducing adipose metabolic derangements and mild chronic inflammatory state [38]. Clinical studies have indicated that increased TC and TG lead to deterioration of glucose tolerance and disorders of glucose metabolism and that a high level of TC can predict T2DM, consistent with our study [36]. In addition, our finding showed that family history of T2DM was a predictive factor of new onset T2DM, which is associated with clear genetic predisposition for T2DM mentioned in previous studies [39].

We built a nomogram to assess the probability of T2DM combining these risk factors. Healthcare workers can make a preliminary judgment on the risk of T2DM in overweight/obese individuals and follow-up with those high-risk populations closely. The high-risk individuals might represent a subset of those who might benefit the most from more frequent evaluations (with FPG and blood lipid detection and weight monitoring). Furthermore, the use of moderate exercise, healthy diet, lipid-lowering therapies, and excess weight loss might be pursued more aggressively for high-risk individuals, which may play a vital role in delaying the onset of diabetes and related complications.

Current study includes several strengths. First, we established a nomogram to predict T2DM for Chinese population with overweight and obesity to make individualized screening possible. Second, our study contains larger sample, multiple study sites, and wider age range, which may merit the data quality and generalizability. It makes the report one of the valuable information for public health sectors and clinical setting. Inevitably, this study has some limitations. First, the study sample is selected from China, which may hamper the representativeness of study results. However, one-fourth of the total people with diabetes live in China, which makes the nomogram significantly useful. Second, our research database is derived from the health check database, so it may bring some deviations to the selection of the study population. For example, the current smoking rate of the sample population in this study is significantly lower than the national average. Third, drug treatment of hypertension and dyslipidemia were associated with an increased risk of new onset diabetes [40]. However, this study failed to include treatment data on hypertension and dyslipidemia. Fourth, although the robustness of our nomogram was examined extensively with internal validation, external validation could not be conducted. Therefore, the further study of the generalizability to overweight and obese populations in other cohort studies is warranted.

## 5. Conclusions

We developed the nomogram as a potentially useful tool to predict T2DM in Chinese with overweight and obese adults based on a multicenter database, which includes six predictors: age, BMI, FPG, TC, TG, and family history. The nomogram shows good discriminative and calibrative ability, which could help healthcare workers and individuals assess the risk of T2DM in overweight and obese populations, and its external evaluation in wider overweight and obese populations is warranted.

## Figures and Tables

**Table 1:** Characteristics of the subjects in the primary and validation cohort.

	Training set (*N* = 58056)	Validation set (*N* = 24882)	Total cohort (*N* = 82938)	*p* value
T2DM (*n*, %)				
No	2151 (96.3)	23964 (96.3)	79869 (96.3)	0.913
Yes	55905 (3.7)	918 (3.7)	3069 (3.7)	
Follow-up (years)	2.98 (2.15–3.93)	2.99 (2.16–3.93)	2.98 (2.15–3.93)	0.141
Age (years)	44.97 ± 12.97	45.02 ± 13.01	44.99 ± 12.98	0.652
Gender (*n*, %)				
Men	42082 (72.5)	17868 (71.8)	59950 (72.3)	0.047
Women	15974 (27.5)	7014 (28.2)	22988 (27.7)	
BMI (kg/m^2^)	26.58 ± 2.24	26.57 ± 2.24	26.57 ± 2.24	0.584
Overweight: 24.0 ≤ BMI < 28.0	45308 (78.0)	19464 (78.2)	64772 (78.1)	0.559
Obesity: BMI ≥ 28.0	12748 (22.0)	5418 (21.8)	18166 (21.9)	
FPG (mmol/L)	5.06 ± 0.63	5.06 ± 0.64	5.06 ± 0.63	0.885
Blood pressure (mmHg)				
SBP	125.25 ± 16.28	125.32 ± 16.39	125.27 ± 16.31	0.587
DBP	78.09 ± 10.99	78.16 ± 11.10	78.11 ± 11.02	0.384
TC (mmol/L)	4.90 ± 0.90	4.89 ± 0.92	4.90 ± 0.91	0.943
TG (mmol/L)	1.77 ± 1.25	1.77 ± 1.28	1.77 ± 1.26	0.663
LDL-c (mmol/L)	2.85 ± 0.68	2.84 ± 0.69	2.85 ± 0.69	0.436
HDL-c (mmol/L)	1.28 ± 0.27	1.28 ± 0.27	1.28 ± 0.27	0.569
ALT (mmol/L)	31.92 ± 26.31	31.74 ± 25.00	31.87 ± 25.92	0.344
AST (mmol/L)	26.76 ± 12.97	26.69 ± 12.13	26.74 ± 12.72	0.479
BUN (mmol/L)	4.85 ± 1.18	4.85 ± 1.19	4.85 ± 1.19	0.760
Creatinine (mmol/L)	74.50 ± 15.61	74.27 ± 15.78	74.43 ± 15.66	0.056
Current cigarette smoking (*n*, %)				
No	52403 (90.3)	22597 (90.8)	75000 (90.4)	0.013
Yes	5653 (9.7)	2285 (9.2)	7938 (9.6)	
Current alcohol drinking (*n*, %)				
No	54173 (93.3)	23179 (93.2)	77352 (93.3)	0.412
Yes	3883 (6.7)	1703 (6.8)	5586 (6.7)	
Family history (*n*, %)				
No	56858 (97.9)	24388 (98.0)	81246 (98.0)	0.466
Yes	1198 (2.1)	494 (2.0)	1692 (2.0)	
Hypertension (*n*, %)				
No	44852 (77.3)	19142 (76.9)	63994 (77.2)	0.306
Yes	13204 (22.7)	5740 (23.1)	18944 (22.8)	
Dyslipidemia (*n*, %)				
No	35689 (61.5)	15235 (61.2)	50924 (61.4)	0.508
Yes	22367 (38.5)	9647 (38.8)	32014 (38.6)	

T2DM, type 2 diabetes mellitus; FPG, fasting plasma glucose; BMI, body mass index; SBP, systolic blood pressure; DBP, diastolic blood pressure; TC, total cholesterol; TG, triglycerides; LDL-c, low-density lipoprotein cholesterol; HDL-c, high-density lipoprotein cholesterol; ALT, alanine aminotransferase; AST, aspartate aminotransferase; BUN, blood urea nitrogen; CCR: concentration of creatinine.

**Table 2:** Risk factors associated with T2DM among overweight and obesity population by Cox proportional hazards regression model

Stratification	Univariate analysis		Multivariate analysis	
	HR (95% CI)	*p* value	HR (95% CI)	*p* value
Age (count)	1.06 (1.04–1.08)	<0.001	1.04 (1.02–1.06)	<0.001
BMI (count)	1.18 (1.08–1.28)	<0.001	1.14 (1.04–1.24)	0.004
FPG (count)	4.60 (3.28–6.46)	<0.001	3.31 (2.28–4.81)	<0.001
TC (count)	1.62 (1.22–2.15)	0.001	1.52 (1.23–2.06)	0.006
TG (count)	1.18 (1.16–1.20)	<0.001	1.07 (1.05–1.10)	<0.001
Family history of T2DM (yes vs. no)	1.41 (1.08–1.82)	<0.001	1.46 (1.12–1.88)	<0.001

The above variables were identified by LAOSSO regression; multiple cox regression adjusted the variables including age (count), BMI, FPG, TC, TG, and family history of T2DM. T2DM, type 2 diabetes mellitus; FPG, fasting plasma glucose; HR, hazard ratio; CI, confidence interval.

## Data Availability

The materials included in the manuscript, including all relevant raw data, will be made freely available to any researchers who wish to use them for noncommercial purposes, while preserving any necessary confidentiality and anonymity.
